# Driving electrochemical corrosion of implanted CoCrMo metal via oscillatory electric fields without mechanical wear

**DOI:** 10.1038/s41598-021-01810-5

**Published:** 2021-11-16

**Authors:** Thomas S. Welles, Jeongmin Ahn

**Affiliations:** Department of Mechanical and Aerospace Engineering, Syracuse University, 727 E Washington St, Syracuse, NY 13244-1240 USA

**Keywords:** Corrosion, Biomedical materials, Implants, Biomedical engineering, Mechanical engineering

## Abstract

Decades of research have been dedicated to understanding the corrosion mechanisms of metal based implanted prosthetics utilized in modern surgical procedures. Focused primarily on mechanically driven wear, current fretting and crevice corrosion investigations have yet to precisely replicate the complex chemical composition of corrosion products recovered from patients’ periprosthetic tissue. This work specifically targets the creation of corrosion products at the metal on metal junction utilized in modular hip prosthetics. Moreover, this manuscript serves as an initial investigation into the potential interaction between implanted CoCrMo metal alloy and low amplitude electrical oscillation, similar in magnitude to those which may develop from ambient electromagnetic radiation. It is believed that introduction of such an electrical oscillation may be able to initiate electrochemical reactions between the metal and surrounding fluid, forming the precursor to secondary wear particles, without mechanically eroding the metal’s natural passivation layer. Here, we show that a low magnitude electrical oscillation (≤ 200 mV) in the megahertz frequency (10^6^ Hz) range is capable of initiating corrosion on implanted CoCrMo without the addition of mechanical wear. Specifically, a 50 MHz, 200 mVpp sine wave generates corrosion products comprising of Cr, P, Ca, O, and C, which is consistent with previous literature on the analysis of failed hip prosthetics. These findings demonstrate that mechanical wear may not be required to initiate the production of chemically complex corrosion products.

## Introduction

Development of modular hip prostheses over the past few decades has allowed for the increased success of total hip arthroplasty (THA), making it one of the most successful surgeries to increase patient mobility and decrease pain^1,2^. The modular hip prosthetic allows the surgeon to select from various sizes of components during surgery to develop a bespoke prosthetic fitment for each individual patient. The modular femoral neck and head require intraoperative assembly, relying on a Morse taper for fixture^3–5^. The Morse taper is a specifically designed self-interlocking taper, free of adhesive or cement. Although the introduction of the Morse taper has allowed for the increase modularity in hip prosthetics, generations of hip prosthetics utilizing this type of modular junction, in which metal is in direct contact with metal, suffer from severe inflammatory reactions of the periprosthetic soft tissue, which lead to chronic pain and/or implant failure^6–8^. This reaction appears to be initiated by the interaction between corrosion products generated at the metal interfaces and macrophages in the periprosthetic tissue. Phagocytosis of corrosion products and macrophage necrosis is accompanied by a perivascular lymphocytic infiltration, which is then followed by soft tissue necrosis^9^. This localized tissue necrosis can become extensive, resulting in injury to abductor muscles and tendons as well as aseptic loosening/osteolysis of the implant-bone interface. This soft tissue and bone damage increases the risk of post revision complications, and frequently results in significant patient morbidity^6,10^.

Current research into the failure of metal interfaces and THA implant corrosion, leading to the inflammatory response and necrosis of periprosthetic tissue, has focused on mechanical wear as the primary mechanism of degradation^11^. Fretting corrosion relies on micro-motion at the modular junction (Morse taper) to erode the protective oxide layer and generate particulate matter that then undergoes a natural galvanic type electrochemical reaction as it contacts the periprosthetic tissue and fluids^12–14^. Crevice corrosion is then capable of acting within the fluid pocket at the eroded junction. Fretting theories indicate that corrosion occurs when the natural passivation/oxide layer, which thickens upon initial implantation of the metal into the body, is broken via mechanical damage^11,14–16^. Crevice corrosion can then accelerate the erosion and embrittlement of the material at the interface between femoral head and modular femoral neck, as the crevice preferentially begins to act as an anodic reaction zone. Although fretting and crevice corrosion are evident at the modular junction, studies often do not accurately replicate the complex chemical composition of wear particles recovered from patients with failed prostheses^17–23^.

This work investigates the potential for an electrically driven, electrochemical corrosion mechanism that occurs concurrently with fretting and crevice corrosion in the implanted environment. The human body is a complex dynamic chemical, electrolytic, electrochemical, and electrically conductive system^24–26^. Therefore, the introduction of a metal based prosthesis into such a system, subjected to constant bombardment of ambient electromagnetic radiation, allows for the potential of electrical charging and discharging at the implant’s surface^27–30^. This work investigates the potential for the interaction between electrical oscillation and the electrochemical reactions occurring at the surface of the implant. Here, we show that such electrical charging and discharging of a typical CoCrMo metal sample within simulated synovial fluid has the ability to initiate a deposition growth on the metal’s surface, by which the base elements of the underlying alloy are reacted into complex chemical species at the surface. This electrochemically driven corrosion can accurately replicate the chemical composition of the corrosion products found near failed implants without requiring mechanical degradation of the metal’s passivation layer. Furthermore, it is demonstrated that if the implanted metal sample is shielded from electrical activity, the deposition matching recovered corrosion products does not appear to occur.

## Method

### Sample preparation

Samples were cut from medical grade ASTM F1537 Alloy 1 round stock to serve as a simulated hip implant. The chemical composition of the alloy is found below in Table 1.

**Table 1:** Elemental analysis of CoCrMo metal alloy used in testing provided by United Performance Metals. Report completed by Carpenter Technology Corporation at time of purchase^31^.

ASTM F1537 Alloy 1 CoCrMo alloy chemical composition by weight percent	
Elements	Weight %
Cobalt	65.05
Chromium	27.76
Molybdenum	5.54
Trace elements	
Carbon	0.05
Manganese	0.79
Silicon	0.59
Phosphorous	0.003
Sulfur	0.005
Nickel	0.04
Copper	0.01
Aluminum	0.04
Nitrogen	0.175
Titanium	0.004
Tungsten	0.02
Boron	0.001
Iron	0.12

The 1″ diameter round stock was crosscut with a diamond cut-off blade which resulted in a 4.5 mm thick disc. The disc was further cut into quadrants with each quadrant receiving a 2.5 mm hole for wire attachment in the future. The samples were then wet sanded to remove any surface imperfections with a Buehler Metaserv 250 grinder/polisher and P400 grit silicon carbide wet polishing paper. P400 grit was chosen to represent surface finish of the prosthetic implant at the modular neck junction. This finish is indicative of the femoral neck junction present on implants, such as the Stryker™ Accolade system^32^. The samples were sanded down to 3.5 mm in thickness to ensure the removal of any cutting marks. The curved face of the samples were further polished with P2500 grit silicon carbide wet polishing paper to develop a high sheen which mimics the polished surfaces of a hip prosthetics found adjacent to the femoral junction. Samples were prepared in this manner to mimic the variety of surface finishes and their proximity to one another in a commercially available prosthetics. The type of surface finish may alter the propagation of electromagnetic activity within the sample and simulated fluid, thus much care was taken to accurately replicate surface finishes.

Each sample was then wired with a medical grade one titanium wire lead, representing the titanium hardware used in the installation of prosthetic hips^33^. The 16 g wire was attached through the hole in each sample specimen. The chemical composition of the titanium wire used is shown in Table 2.

**Table 2:** Elemental analysis of titanium grade 1 wire used in testing provided by TEMCo Industrial^34^.

ASTM F76 Ti Grade 1 chemical composition by weight percent	
Elements	Weight %
Titanium	≥ 99.6
Trace elements	Maximum weight %
Nitrogen	0.03
Carbon	0.08
Hydrogen	0.01
Iron	0.20
Oxygen	0.18
Maximum total residuals	0.40

Although titanium alloys are often preferred in the orthopedic industry, commercially pure titanium was selected for use as the electrical conductor in this study. Commercially pure titanium is reported to exhibit equal, or potentially greater resistance to corrosion than its alloys^35,36^. Moreover, the enhanced mechanical properties obtained by alloying titanium were not required for this study.

Two of the prepared samples were each placed into a sterilized Pyrex petri dish and subsequently submerged in 100 mL of simulated synovial fluid. Each wire lead exited the side of the petri dish under the lid. The samples were positioned such that they were 1 mm apart within the simulated synovial fluid test solution. The simulated synovial fluid was a 1:1 (by volume) mixture of bovine serum (BS), purchased from Millipore Sigma™ (Prod No. 12306C), and de-ionized (DI) water. Bovine serum and deionized water has been previously utilized in published studies as an adequate surrogate of synovial fluid^7,11–16,37–39^. The exact chemical composition of the BS is unknown, but it is known to contain ≤ 10.00 EU/ml of endotoxin, 3.0–4.5 g/dL total protein, ≤ 25 mg% hemoglobin, and to have a PH of 6.8–8.1. BS is a complex mixture of salts, proteins, and lipids. Any elements identified during testing which are not reported in the metal samples or in the preparation of samples, is assumed to be a result of the BS (Table 3).

**Table 3:** Typical components found in BS.

Chemical species	Average	Range	Chemical species	Average	Range
Protein (mg/ml)	38	32–70	Interleukins		
Albumin (mg/ml)	23	20–36	Interferons		
Hemoglobin (μg/ml)	113	24–181	Free and protein-bound fatty acids		
α1- Antitrypsin			Triglycerides		
α2-Macroglobulin			Phospholipids		
Transferrin			Cholesterol (μg/ml)	310	120–630
Transcortin			Ethanolamine		
α1-Lipoprotein			Phosphatidylethanolamine		
β1-Lipoprotein			Retinol/retinoic acid (Vitamin A) (ng/ml)	90	10–350
Fibronectin			Thiamine		
Laminin			Riboflavin		
Serum spreading factor			Pyridoxine/pyridoxalphosphate		
Lactate dehydrogenase			Cobalamin		
Alkaline phosphatase			Folic Acid		
γ-Glutamyl			Niacinamide/nicotinic acid		
Transferase			Panthotenic Acid		
Alanine			Biotin		
Insulin (μU/ml)	10	6–14	Ascorbic acid (Vitamin C)		
Cortisol (ng/ml)	0.5	0.1–23	α-Tocopherol (Vitamin E) (ng/ml)	1.1	1–4.2
Vasopressin			Glucose (mg/ml)	1.25	0.85–1.81
Thyroid hormones (ng/ml)	1.22	0.2–4.5	Galactose		
Parathyroid hormone (ng/ml)	1.72	0.085–6.18	Fructose		
Growth hormone (ng/ml)	39.0	18.7–51.6	Mannose		
Pituitary glandotropic factors			Ribose		
Prostaglandin E (ng/ml)	5.9	0.5–30.5	Glycolytic metabolites		
Prostaglandin F (ng/ml)	12.3	3.8–42.0	Urea (μg/ml)	160	140–200
Epidermal growth factor			Purines/pyrimidines		
Fibroblast growth factor			Polyamines		
Nerve growth factor			Creatinine (μg/ml)	31	16–43
Endothelial cell growth factor			Amino Acids		
Platelet-derived growth factor			Bilirubin (μg/ml)	4	3–11

Averages and range estimates of concentrations given when available.

*Note that the exact composition of BS is unknown, the above table provides only estimates of concentrations and constituents^40,41^.

### Corrosion testing procedure

**Figure 1:**
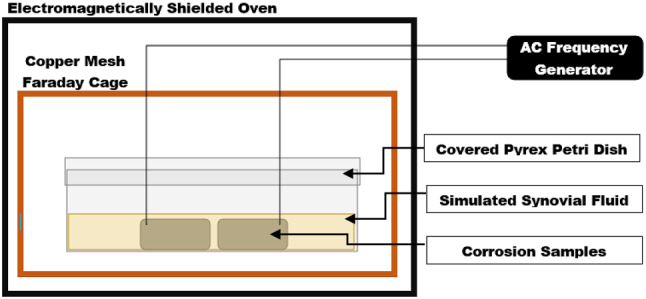
Schematic of corrosion experimental setup.

The samples subjected to electrical activity were connected to a Rigol™ DG2000 frequency generator with 16 bit resolution, connected to an Ametek™ power conditioner/stabilizer, for simulated electrical oscillation. A bacteria culture of the simulated synovial fluid was taken at the start and end of corrosion testing for each test specimen. The bacteria culture was used to identify any bacterial contamination of test results. No fluid sample exhibited bacterial growth at the start or end of testing. The test specimens were then placed into a faraday cage, with a 0.006 in copper mesh weave and signal barriers between the test specimens, within an electrically shielded incubation oven at 37 °C for 90 days. For each test condition, two samples were subjected to electrical oscillations and two baseline sample was shielded from electrical oscillation. Therefore, each test condition resulted in two samples which ensures repeatability, as well as two baseline samples. A schematic of the experimental setup may be seen in Fig. 1.

### Test conditions

Three test conditions were investigated for electrochemically driven corrosion of implanted CoCrMo. Test condition A represents the baseline for corrosion samples shielded from any electrical activity during the duration of the test. The samples under test condition B were subjected to a 50 MHz sine wave with a peak-to-peak potential (Vpp) of 200 mVpp, centered at 0 V. The samples under test condition C were subjected to a 25 MHz square wave at 200 mVpp, centered at 0 V. Samples of all test conditions were placed within the same batch of simulated synovial fluid. The voltage magnitude was selected in accordance to previously published literature for recorded interference in nerve conduction studies, skin electrode data, and experimental work on biological effects of external electric fields^42–46^. The frequency range was selected to replicate the oscillations that could develop on implanted metal from the highly active communications spectrum^47–51^. Condition B represents a single frequency response, whereas condition C was selected to illustrate corrosion behavior under excitement of multiple frequency harmonics, not present in condition B. The operating conditions are summarized in the following Table 4.

**Table 4:** Operating variables and test conditions.

Test condition	Condition A	Condition B	Condition C
Number of samples tested	2	2	2
Corrosion fluid	1:1 Ratio by volume of Millipore Sigma™ BS 12306C and DI-Water		
Electrical oscillation frequency	No electrical activity sourced	50 MHz	25 MHz
Wave shape		Sinusoidal	Square
DC Offset		0	0
AC oscillation amplitude		200 mVpp	200 mVpp

All samples from conditions A, B, and C will henceforth be referred to simply as Sample A, B, and C, respectively. The results shown are indicative of all samples tested at each condition. Therefore, the results shown are repeatable for each testing condition and represented below in a concise and succinct manner.

### Electrochemical impedance spectroscopy testing

During the duration of the lab testing, the samples subjected to electrical oscillations were connected to a Solartron™ 1287/1260 electrochemical impedance spectroscopy (EIS) suite. An EIS response frequency sweep, at 10 mV amplitude, was performed, using Zplot™, on each specimen from 1.5 × 10^6^ Hz to 5.0 × 10^−2^ Hz at 10 mV, to characterize the electrochemical properties of each specimen. The EIS frequency sweep tests were performed in the original test simulated synovial fluid and in fresh simulated synovial fluid at the conclusion of the test. In addition, cyclic voltammetry, at a sweep rate of 100 mV/sec from—0.8 V to 1.4 V, was performed on all samples in new simulated synovial fluid with the conclusion of testing using Corrware™ software in conjunction with the Solartron™ EIS suite. All test equipment was powered through an Ametek™ power conditioner/stabilizer to prevent electrical building noise from interfering with the test samples. For all EIS experiments, a 2-probe method was utilized to prevent contamination of the simulated synovial fluid testing medium. Utilization of the 2-probe method allowed for each metal sample to act as an electrode without introducing any other material into the simulated synovial fluid. As such, no tertiary reference probe was utilized. The EIS measurements utilized an ideally symmetric cell, and therefore a reference of 0 V was utilized as the center point of measurements.

### Scanning electron microscopy procedure

At the conclusion of each test, the samples were removed from the testing solution and rinsed with DI water. The samples were then gently wiped clean with Kimwipes™ to remove any loose material. Following this, the samples were rinsed again with DI water, dried, and bagged for analysis. Samples were rinsed in DI water, instead of a more caustic cleaning agent, to prevent the disruption of the organic film on the metals surface. It was believed that analysis with the organic film intact may indicate potential reaction mechanism found within an in vivo setting^52–54^. Although, there may be risk of bacterial contamination when clearing the samples with DI water, no bacterial culture indicated contamination. Each sample was affixed to the scanning electron microscope (SEM) fixture via conductive carbon adhesive ink. The samples then underwent visual inspection under the SEM as well as an energy dispersive x-ray spectroscopy (EDS) analysis. The SEM used was a JEOL™ JSM-IT100 capable of 33–300,000X magnification with EDS capability.

### X-ray diffraction analysis procedure

After SEM analysis the samples were further analyzed via x-ray diffraction (XRD) with a Bruker™ D2 Phaser XRD system. Each sample was placed into a 4.5 mm deep well XRD sample holder. Each sample was mounted in the sample holder such that the sample surface was level with top of the sample holder.

### Laser ablation inductively coupled mass spectroscopy analysis procedure

Furthermore, 5 ml of each test solution was placed onto a glass slide and allowed to dry. A Teledyne™ CETAC Analyte Excite Excimer Laser Ablation System was then used to analyze each dried solution sample for Cr ^Isotopes 50–54^, Co^Isotope 59^, and Mo^Isotopes 92–100^. A background for each signal was calculated using 26 separate measurements for each sample. Next, each signal was recorded and averaged using 330 separate measurements for each sample. The background was then subtracted from the signal to give the relative counts per second of each isotope. Standard error was calculated for each signal.

## Results and discussion

**Figure 2:**
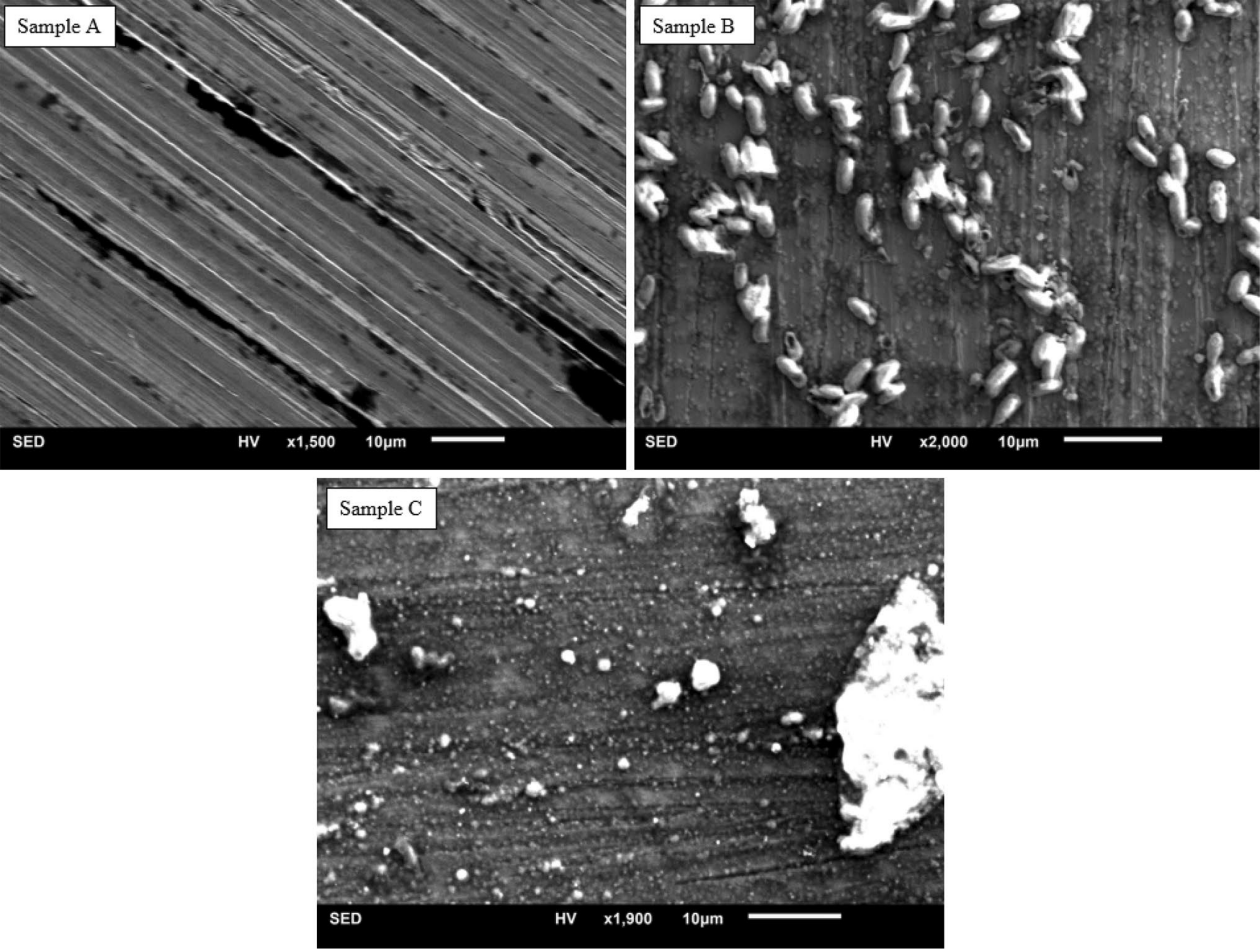
Scanning electron microscope (SEM) secondary electron detector (SED) image of test samples. Sample A, SED-SEM image of Sample A at × 1,500 magnification. Sample A represents the baseline sample, shielded from electromagnetic radiation and electrical oscillation, held in simulated synovial fluid (50/50% by weight deionized water and Millipore Sigma Fetal Bovine Serum). Sample A illustrates only minor surface change from the original polished surface. Sample B, SED-SEM image of Sample B at × 2,000 magnification. Sample B represents a corrosion sample subjected to a 50 MHz sine wave at 200 mVpp held in simulated synovial fluid. Sample B shows significant surface modification, marked by patterned oblong type surface growth. Sample C, SED-SEM image of Sample C at × 1,900 magnification. Sample C represents a corrosion sample subjected to a 25 MHz square wave at 200 mVpp held in simulated synovial fluid. The surface shows significant surface modification, marked by random deposition.

**Figure 3:**
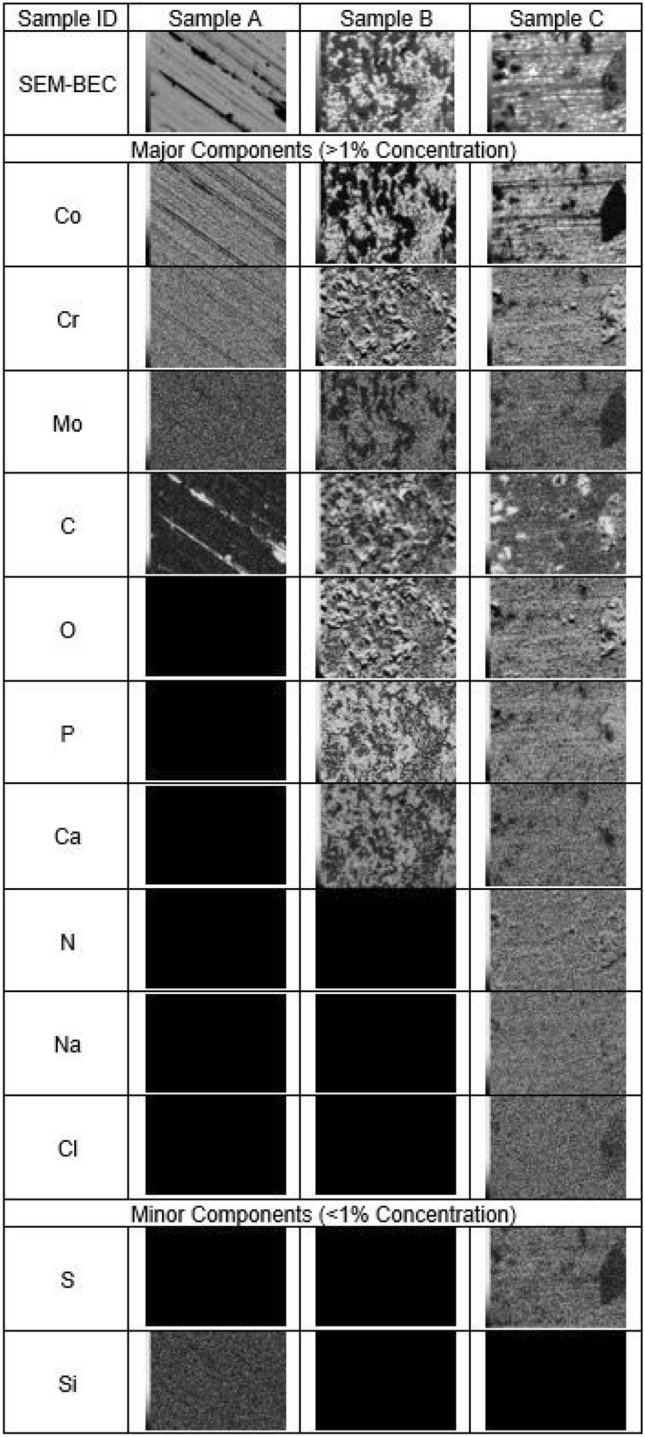
EDS analysis and back scatter electron composite images. Grayscale mapping of surface elemental compositions with back scatter images shown in the first row. Darker shades represent lower concentrations of the specified element, whereas lighter shades indicate higher concentrations of the specified element. Sample A, shielded from electrical activity, shows little elemental change from the base alloy. Sample A contains Co, Cr, Mo, C and traces of Si. Sample B, subjected to a 50 MHz, 200 mVpp sine wave, shows significant surface modification. Sample B contains Co, Cr, Mo, C, O, P, and Ca. Sample C, subjected to a 25 MHz, 200 mVpp square wave, shows the greatest variation in elemental composition. Sample C contains Co, Cr, Mo, C, O, P, Ca, N, Na, Cl, and S.

Analysis via SEM illustrates significant surface modification on all samples subjected to electrical oscillation, Samples B and C, and only minor surface modification on samples shielded from electrical activity, Sample A. Sample A maintains the base metal alloy with surface striations developed during polishing. The surface shows slight addition of dark, non-distinct spotting as seen in Fig. 2A. Sample B, subjected to a 50 MHz sine wave, exhibits significant surface deposition resulting from the interaction of the metal’s surface with simulated synovial fluid. In Fig. 2B, the polishing striations are clearly diminished in comparison to Sample A. The surface has developed well-defined oblong-like structures on the order of ~ 5 µm across the surface; the depositions appear to largely align with the remaining polishing striations. These depositions, though similar in shape to bacteria, are believed to be the result of the electrical activity. No bacterial culture showed contamination throughout testing. All samples of test Condition A display similar growths. Additionally, the surface growths contain significant amounts Cr, as seen in Fig. 3B.

Although Sample C (Fig. 2C) also exhibits significant surface modification, the type of surface modification is markedly different from that depicted by Sample B. The additional frequency harmonics within the square wave significantly modified the type of surface/fluid interaction. Sample C shows indiscriminate surface deposition with variability in size, shape, and orientation. In addition, Sample C shows the most widespread, general surface deposition. As such, the striations from polishing are nearly indistinguishable.

The surface deposition identified in Fig. 2 was further analyzed for elemental composition via energy dispersive x-ray spectroscopy (EDS) is shown in Fig. 3.

The samples shielded from electrical activity, Sample A, appears to be predominately composed of the base CoCrMo alloy, consistent with the manufacturing standard provided in the Method Section. The darkened areas highlighted in Fig. 2 are identified as C deposits arising from the simulated synovial fluid. The micrographs of Sample B illustrate a distinct pattern of elemental composition present in the surface deposition growths. The area void of surface deposition is the base CoCrMo alloy. However, within the surface deposition, there are significant amounts of Cr, C, O, P, and Ca. The clear pattern presents within the Cr micrograph, matching that of C, O, P, and Ca, indicates that Cr is releasing from the base metal and interacting with the simulated synovial fluid to form the surface particles. The incorporation of additional frequency harmonics, in Sample C, introduced a higher degree of complexity to the surface deposition. Sample C contains N, Na, and Cl, in addition to C, O, P, Ca, Cr, Co, and Mo. Surface deposition on Sample C shows mixed composition. The large growth on the right of Sample C’s micrographs in Fig. 3 shows a clear combination of Cr, C and O, with trace amounts of N, whereas other surface growths contain Cr and O, without C. Therefore, Sample C is characterized as a general surface deposition in comparison to Samples A and B. In Sample C, the entirety of the surface illustrates an elemental composition change. In comparison, the surfaces of Samples A and B contain only base metal elements outside of the surface growths.

**Figure 4:**
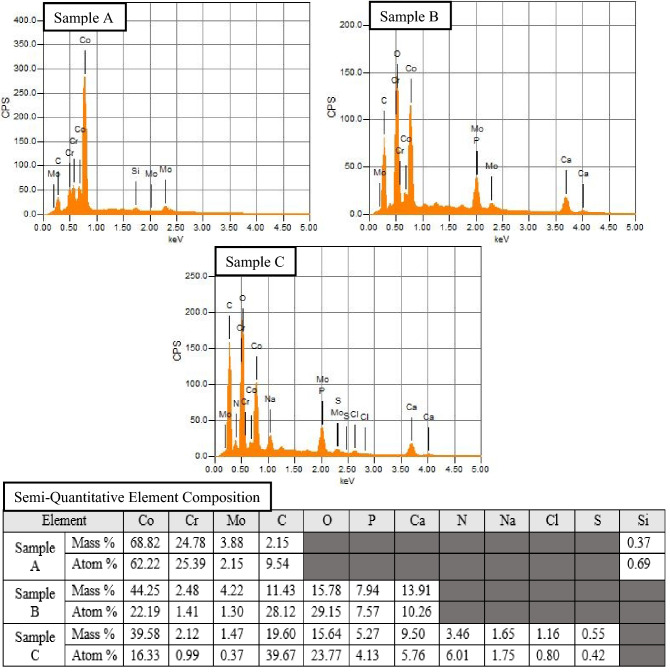
Energy spectrum images and semi- quantitative elemental analysis table from EDS. Elemental spectrum and semi-quantitative analysis arising from EDS analysis. Sample A, shielded from electrical activity, shows little elemental change from the base alloy. Sample B, subjected to a 50 MHz, 200 mVpp sine wave, shows significant surface modification. Sample C, subjected to a 25 MHz, 200 mVpp square wave, shows the greatest variation in elemental composition. Semi-Quantitative Element Composition table shows the mass percentage and atom percentage composition of each element arising from the energy spectrum. *Note: quantitative EDS may not be accurate for light elements such as C and O, therefore in the manuscript it is labeled as semi-quantitative.

Further, semi-quantitative EDS analysis was completed to identify relative abundance of elements beyond basic greyscale mapping. The spectrums and results of the semi-quantitative EDS are given in Fig. 4.

The semi-quantitative EDS spectrum of Sample A nearly matches that of the base CoCrMo alloy. As shown in Fig. 4, Sample A consists of ~ 68 wt% Co, ~ 25 wt% Cr, ~ 4 wt% Mo, 0.37 wt% Si, with only ~ 2 wt% additional C from the synovial fluid. Samples B and C, in stark contrast to Sample A, show that the majority of the surface has been altered into corrosion products. Sample B shows ~ 11 wt% C, ~ 16 wt% O, ~ 8 wt% P, and ~ 14 wt% Ca resulting from the interaction with simulated synovial fluid. The spectrum of Sample B is consistent with previously published retrieval studies of in vivo corrosion species^8,20,22,55,56^. Urban et al. reported the analysis of retrieved wear particles consisted primarily of chromium oxides and chromium phosphates, with varying degrees of Ca and Co present^57^. This 1994 finding was further substantiated by Hart et al.^58^ in 2010, Xia et al.^22^ and Oskouei et al.^20^ in 2017, and Eltit et al.^8^ in 2019, in which all report significant findings of chromium oxides and chromium phosphates in the secondary wear particles of implanted CoCrMo.

The addition of N, Na, and Cl across the surface of Sample C is non-negligible (elemental detection of greater than 1 wt%). Therefore, the results from EDS indicate the potential for multiple corrosion responses: 1. the formation of distinct, uniform crystalline deposition which could, when subjected to mechanical wear, form secondary wear particles, as seen in Sample B; and 2. generalized modification of the surface chemical composition.

**Figure 5:**
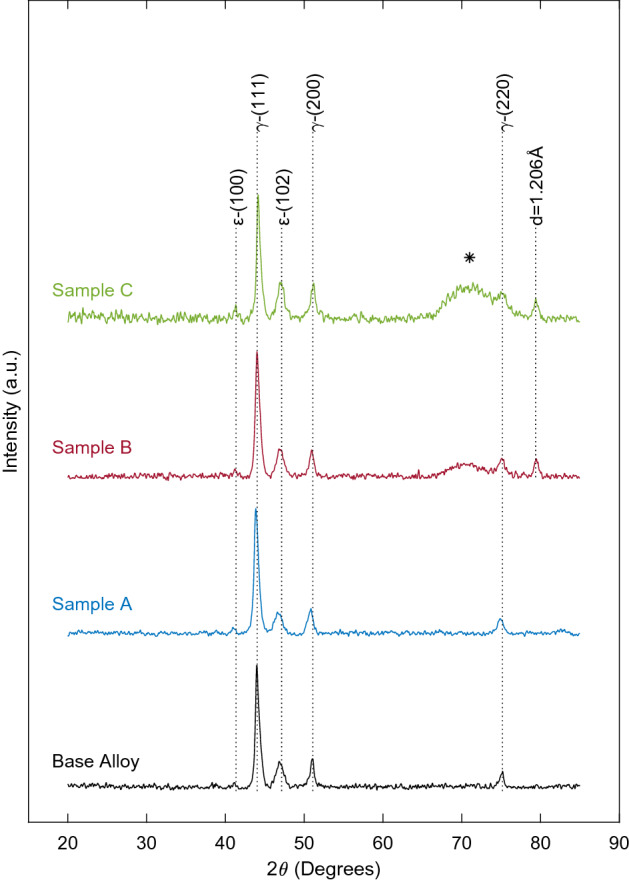
XRD analysis of test samples. Base Alloy, representing an untested sample, displays a diffraction pattern with peaks at 2θ equal to 41.3°, 44.1°, 47.1°, 51.0°, and 75.1°. Sample A, shielded from electrical activity, displays a diffraction pattern with peaks at 2θ equal to 41.3°, 44.1°, 47.1°, 51.0°, and 75.1°. Sample B, subjected to 50 MHz 200 mVpp sine wave, displays a diffraction pattern with peaks at 2θ equal to 41.3°, 44.1°, 47.1°, 51.0°, 75.1°, 79.3°, and a broad peak between ~ 69°–71°. Sample C, subjected to 100 MHz 200 mVpp square wave, displays a diffraction pattern with peaks at 2θ equal to 41.3°, 44.1°, 47.1°, 51.0°, 75.1°, 79.3°, and a large, broad peak between ~ 69°–78°.

In order to identify the crystallographic structure present in the surface deposits and underlying metal alloy, the samples were investigated with x-ray diffraction spectroscopy (XRD), shown in Fig. 5.

The spectrum labeled Base Alloy, representing an untested sample of ASTM F1537 CoCrMo alloy, displays a diffraction pattern with peaks at 2θ equal to 41.3°, 44.1°, 47.1°, 51.0°, and 75.1°. The diffraction pattern is consistent with prior published work^41^, identifying the majority of the material as a face centered cubic (FCC) γ-(Co, Cr, Mo) crystallographic structure indicated by major peaks at 44.1° and 51.0°. Lesser peaks at 41.3° and 47.1° indicate a small amount of hexagonal close pack (HCP), ε-(Co, Cr, Mo) crystallographic structure, labeled in Fig. 5^59,60,61^.

The XRD diffraction pattern for Sample A, shielded from electrical activity within simulated synovial fluid, shows no significant change when compared to the base alloy. Therefore, it is concluded that no crystallographic structure change occurred in the metal alloy within the simulated synovial fluid over the three-month test duration when shielded from electrical activity.

**Figure 6:**
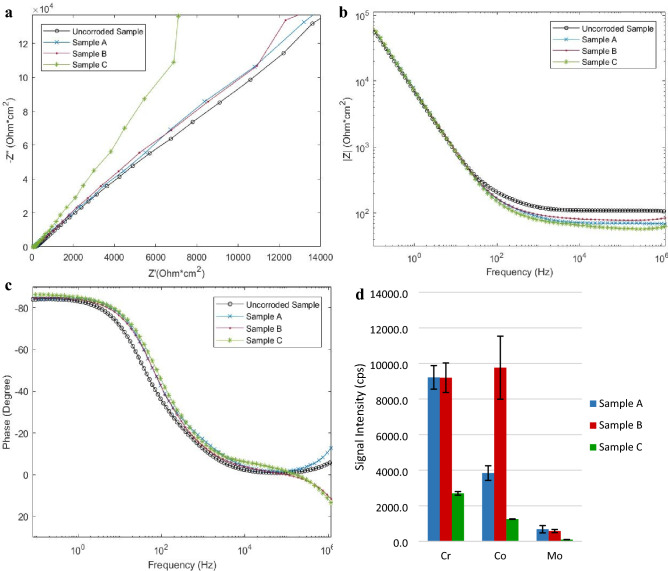
Area normalized Electrochemical impedance spectroscopy (EIS) analysis and relative metal ion concentration in the simulated synovial fluid at test completion. EIS analysis of the corroded samples in original test simulated synovial fluid. The uncorroded sample represents a freshly prepared, non-corroded test specimen. Sample A was shielded from electrical activity, Sample B was subjected to a 50 MHz, 200 mVpp sine wave, and Sample C was subjected to a 25 MHz, 200 mVpp square wave. (**a**) Comparison of positive real impedance (x-axis) and negative imaginary impedance (y-axis). (**b**) Total magnitude of impedance (y-axis) is plotted against frequency (x-axis). (**c**) Phase angle (y-axis) is plotted against frequency (x-axis). (**d**) Base metal ion concentration intensity in the simulated synovial fluid after testing from laser ablation inductively coupled plasma mass spectroscopy (LAICPMS).

Investigation of samples under oscillatory electrical fields, Samples B and C, indicate a significant departure from the original diffraction pattern. Consistent with *in-vivo* corrosion product analysis from retrieval studies, the samples show the original diffraction pattern with the creation of additional peaks^20,22^. Both Samples B and C display the original diffraction pattern with the creation of a broad peak, labeled as *, beginning at 2θ ~ 69° followed by a narrow peak at 2θ = 79.3°, labeled with interplane spacing d = 1.206A. The crystallographic change is believed to result from a combination of amorphous deposition, represented by the broad peak labeled as *, as well as the creation of crystalline corrosion products and/or oxidation state change within the metal sample, represented by the creation of the peak at 2θ = 79.3°, d = 1.206A. The broad peak beginning at 2θ ~ 69° is substantially larger in magnitude and breadth in Sample C, when compared to Sample B. This finding is consistent with a primarily amorphous surface deposition theorized in the above EDS analysis. However, Sample C does display crystalline deposition or crystalline surface modification indicated by the addition of a sharp peak at d = 1.206A. Conversely, in Sample B, the amorphous peak is substantially lower in magnitude. This, in conjunction with a strong sharp peak at an interplane spacing of d = 1.206A, suggests only minor amorphous surface deposition, yet significant interaction of the crystalline structure of the sample with the test solution and/or suggests that the surface deposits are crystalline in nature. This could indicate the potential for a greater release of metal ions into solution of simulated synovial fluid, which is investigated in Fig. 6d.

In order to identify, understand, and potentially predict this electrochemical corrosion mechanism more holistically, the electrochemical behavior change and metal ion release over the test duration was investigated, as shown in Fig. 6.

The most notable change seen in Fig. 6 is the decrease in total impedance from baseline for all test samples, shown in Fig. 6b, at frequencies above 10^2^ Hz and the divergent behavior in phase angle above 10^5^ Hz for Samples B and C. Sample C displays moderately greater capacitive behavior, as seen in Fig. 6a,c, when compared to the other samples. These characteristics are believed to result from the interaction of dissolved metal ions released into solution and the corrosion products identified on the surface of the material. Therefore, the simulated synovial fluid used during testing was analyzed for Cr, Co, and Mo content for each test condition via LAICPMS. Prior to testing the simulated synovial fluid did not contain Cr, Co, or Mo. All three test conditions displayed metal ion release into solution, which is consistent with the decrease in overall impedance for all samples at high frequency. However, Sample B shows disproportionately higher concentrations of Co. Therefore, it is theorized that during the growth of Cr, C, O, Ca, P crystals on Sample B, Cr is removed from the base metal and drawn into the surface crystal. The removal of Cr from the base metal destabilizes Co within the metal lattice, allowing it to more readily release into the simulated synovial fluid. The elevated levels of Co within the fluid are consistent with reported patient data where prosthetic failures have occurred^62^. In comparison, Sample C displays the lowest overall metal ion release into solution. In conjunction with the previous EDS and XRD analysis, it appears as though Sample C is simply attracting material to the surface without releasing metal ions into solution, thus supporting the previous hypothesis of generalized surface deposition.

**Figure 7:**
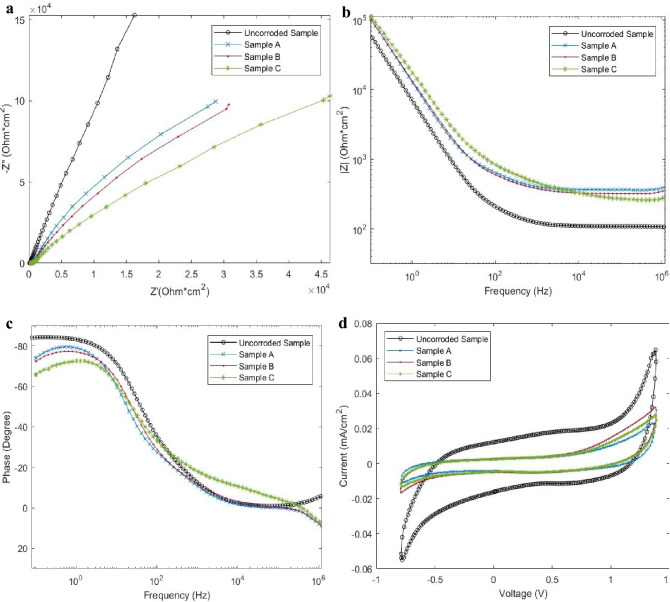
Area normalized EIS analysis and cyclic voltammetry analysis of corroded samples in fresh simulated synovial fluid. EIS analysis of the corroded samples in freshly prepared simulated synovial fluid. The uncorroded sample represents a freshly prepared, non-corroded test specimen. Sample A was shielded from electrical activity, Sample B was subjected to a 50 MHz, 200 mVpp sine wave, and Sample C was subjected to a 25 MHz, 200 mVpp square wave. (**a**) Comparison of positive real impedance (x-axis) and negative imaginary impedance (y-axis). (**b**) Total magnitude of impedance (y-axis) is plotted against frequency (x-axis). (**c**) Phase angle (y-axis) is plotted against frequency (x-axis). (**d**) Cyclic voltammetry analysis of samples at 100 mv/sec sweep.

The samples were placed into fresh simulated synovial fluid to characterize the electrochemical behavior response without the presence of leached metal ions, Fig. 7.

All samples show increased overall impedance and increased real impedance, Fig. 7b, consistent with the addition of corrosion products and surface passivation from time spent in solution^13,60,63^. All samples show increased inductive/resistive behavior at low frequencies, as indicated by the phase angle shift toward 0 (Fig. 7b,c). Samples A and B show similar impedance behavior and Sample C shows significant deviation from all corroded and uncorroded samples. Each sample was then tested via cyclic voltammetry (CV) to demonstrate the current response of the active reduction/oxidation system. All samples display a decrease in the magnitude of the current response to the applied voltage, when compared to a freshly prepared sample. Sample A and C display similar responses of current, whereas Sample B displays the greatest magnitude of induced current for all reductive and oxidative reactions.

Introduction of the metal sample into the electrolytic simulated synovial fluid creates a natural galvanic response where the reactivity of the metal is passivated by the release of surface ions and the thickening of a carbacious and/or naturally occurring oxide layer^52,64,65^. CoCrMo has previously been reported to decrease electrochemical galvanic activity overtime in an in vitro setting^64,66,67^. This natural response is clearly evident in the behavior and characterization of Sample A. Sample A released the expected metal ions into solution, displayed decreased activity on CV, and showed only minor surface modification. The introduction of electrical oscillation, however, greatly altered the behavior and characterization of the test samples.

Sample B, subjected to a 50 MHz sine wave, displays significantly higher conductivity during the CV testing, Fig. 7d, and at low frequencies during the impedance sweep, Fig. 7b. Sample B resembles Sample A in terms of capacitive behavior and phase angle behavior, Fig. 7a,c. However, Sample B shows decreased total impedance, Fig. 7b,d. The well-defined crystals that have developed on the surface of Sample B significantly increase the overall surface area of the sample, without blocking the base the metal from reacting with the fluid. The disproportionately high concentration of Co within the serum, Fig. 6d, in conjunction with the overall increased conductivity in CV, Fig. 7d, indicate that electrical excitation induced on Sample B is capable of preventing the formation of a passivated layer, simultaneously encouraging the growth of Cr, C, O, Ca, P crystals. This result represents an area not currently addressed in conventional theory. No mechanical wear has been required to remove a passivated layer, yet Sample B shows increased electrochemical activity.

Sample C, subjected to a 25 MHz square wave, represents a distinct departure from anticipated trends. The phase angle response, Fig. 7c, is drastically different across the frequency spectrum. Sample C shows greater inductive characteristics below 100 Hz and greater capacitive like characteristics above 100 Hz. Samples show greater real impendent behavior at low frequency and decreased total impedance at high frequency, Fig. 7a,b, yet Sample C shows marginally higher conductivity in CV testing, when compared to Sample A. The variation in electrochemical behavior is believed to result from the complex surface topography and chemical composition identified previously. The unique surface deposition shown on Sample C results in a dynamic, frequency dependent, electrochemical response. The widespread, generalized surface deposition acts to decrease, or partially block electrochemical activity, while the creation of many small surface crystals within the surface deposition acts to simultaneously increase surface area.

**Figure 8:**
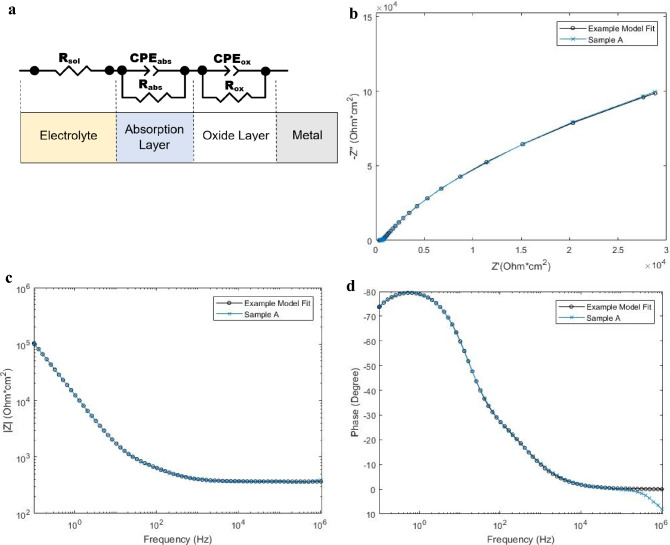
EIS equivalent circuit model fitting example graphs. EIS equivalent circuit modeling for samples analyzed as a symmetric cell in freshly prepared model synovial fluid. (**a**) Equivalent circuit model. R_sol_, solution resistance/solution interaction resistance with absorption layer, R_abs_, absorption/outer layer resistance, CPE_abs_, constant phase element of the absorption/outer layer, R_ox_, oxide/inner layer resistance, CPE_ox_, constant phase element of the oxide/inner layer. (**b**) Comparison of positive real impedance (x-axis) and negative imaginary impedance (y-axis). (**c**) Total magnitude of impedance (y-axis) is plotted against frequency (x-axis). (**d**) Phase angle (y-axis) is plotted against frequency (x-axis).

In order to better quantify the changes illustrated in the EIS analysis, an equivalent circuit model was developed and applied to the system within the original test fluid as well as freshly prepared simulated synovial fluid. The equivalent circuit model is shown in Fig. 8, below. The equivalent circuit model relies on a modified Randle’s type circuit, utilizing Constant Phase Elements (CPE) in place of the capacitor and Warburg diffusion element, for the corrosion of metal alloys. As shown in Fig. 8a, the model is constructed from the metal alloy (right) toward the solution (left). The model assumes a CPE in parallel with a resistor, representing the passivated oxide layer of the CoCrMo alloy. This is then connected in series with a similar modified Randle’s circuit to represent an absorption/outer layer of the metal’s surface. This is then connected in series to a resistor, representing the resistance of the surrounding solution and interaction resistance between the absorption layer and the surrounding solution^38,68^. The model parameters were fit using Zview™ with all variables set to be free and positive. Therefore, the goodness of fit test, χ^2^, is based on (n-1) = 6 degrees of freedom, DOF, as each resistor has one DOF and each CPE has 2 DOF’s. For 6 DOF’s a χ^2^ value of < 0.676 provides a confidence level of 99.5%. As illustrated in Table 5, all models have high goodness of fit. All χ^2^ values are < 0.002. Figure 8b–d are provided as an example of the model’s fit to the experimental data. Figure 8b–d illustrate the model fit to Sample A in freshly prepared synovial fluid. Only one model is illustrated to prevent redundancy, yet all models illustrate similar fit.

**Table 5:** Equivalent circuit parameters of CoCrMo under the studied corrosion environments as symmetric cells.

	R_sol_ (Ω cm^2^)	R_abs_ (Ω cm^2^)	CPE_abs_ (μF/cm^2^)	n_abs_	R_ox_ (MΩcm^2^)		CPE_ox_ (μF/cm^2^)	n_ox_	χ^2^
Uncorroded Sample	108.3	100.9	102.8	0.711		7.539	26.15	0.942	0.00022
**In corrosion simulated synovial fluid**									
Sample A	69.53	60.95	86.97	0.712		10.96	24.18	0.947	0.0013
Sample B	79.26	68.70	247.2	0.626		5.464	24.20	0.946	0.0013
Sample C	56.84	117.9	1183.4	0.421		7.114	23.90	0.959	0.00093
**In freshly prepared simulated synovial fluid**									
Sample A	366.3	291.7	17.18	0.793		6.405	15.69	0.907	0.00062
Sample B	232.6	205.7	21.56	0.746		6.942	15.88	0.880	0.00056
Sample C	256.5	452.8	39.86	0.531		5.472	14.03	0.827	0.00098

R_sol_, solution resistance/solution interaction resistance with absorption layer, R_abs_, absorption/outer layer resistance, CPE_abs_, calculated capacitance of the constant phase element for the absorption/outer layer, R_ox_, oxide/inner layer resistance, CPE_ox_, calculated capacitance of the constant phase element for the oxide/inner layer, n, the exponent of the CPE.

Table 5 is to be interpreted along with Fig. 8. Table 5 represents the model fit parameters as well as the calculated capacitance for each CPE. Capacitance calculations were evaluated following the methods laid forth by V.D. Jovic on behalf of Gamry Insturments^TM^^69^. All samples are expected to demonstrate the addition of organic based films on the surface of the metal, yet the equivalent circuit model will be utilized to elucidate differences between test conditions.

When analyzing the model fit parameters for the samples within the original testing fluid, Table 5 shows that all samples displayed similar values of capacitance for the oxide layer of the material. Sample A, shielded from electrical activity, shows a significant increase in R_ox_, and a minor decrease in CPE_ox_. This behavior could be indicative of a thickening of the passivated oxide layer on the metal. This is in stark contrast to Sample B, displaying a significant decrease in R_ox_, and Sample C which shows a minor decrease in R_ox_. Samples B and C demonstrate significantly higher CPE_abs_ when compared to Sample A and the Uncorroded Sample, which could indicate the potential for increased charge concentration at the absorption layer and thus increase the activity of the metal. R_sol_ decreased for all Samples, as metal ion concentration increased within the solution during testing.

Investigating the model fit of the samples in freshly prepared synovial fluid, all samples indicate similar R_ox_, closely resembling that of the uncorroded sample. All test samples display similar CPE_ox_ values, slightly decreased from the uncorroded sample. The pattern of increased outer layer capacitance, CPE_abs_, of Samples B and C remains in fresh fluid when compared to Sample A, yet all samples indicate a lower CPE_abs_ value in fresh fluid when compared to the uncorroded/untested samples. All test samples have higher R_sol_ when compared to the uncorroded sample, however, Sample B and C have significantly smaller values of R_sol_ when compared with Sample A. The lower R_sol_, in conjunction with increased CPE_abs_, could indicate that Samples B and C have higher activity within the serum when compared to Sample A.

For the above equivalent circuit models, Sample A consistently has greater values of the CPE exponent, n, for both the oxide and absorption layers. Samples B and C, those that were tested with electrical oscillations, indicate n values nearer to 0.5. A value of n = 0.5 in a CPE is representative of a classical Warburg diffusion element, a value of n = 1 is representative of an ideal capacitor, and a value of n = 0 is representative of a resistor. Therefore, it is possible that Samples B and C demonstrate increased diffusion interaction between the solution and the metal. The trends within the model fitting parameters appear to be consistent with the results shown in Figs. 6 and 7.

## Conclusions

This work represents potentially foundational evidence in the generation of corrosion products of implanted metal alloys, not currently addressed in conventional theory. The development of corrosion products presented here required no mechanical wear. The simple manipulation of oscillatory electric fields surrounding the in vitro samples, at magnitudes and frequencies comparable to those resulting from ambient electromagnetic radiation, created and replicated the corrosion products identified on recovered hip prosthesis. Introduction of oscillatory electric fields not only generated corrosion products but also demonstrated that the chemical composition of such products may depend on the frequency of electric excitation.

Shielding the samples from electrical activity, represented by Sample A, prevented the development of corrosion products on the surface of the CoCrMo alloy. These samples showed only minor C deposition from the simulated synovial fluid. The XRD analysis of samples indicated no crystallographic structure change when compared to untested samples of the same metal stock. These findings are consistent with initial studies into the biocompatibility of CoCrMo alloys^66,67^.

Sample B, representing samples subjected to a 50 MHz, 200 mVpp sine wave, showed significant surface modification. The EDS analysis indicated the creation of ordered surface growths consisting of Cr, C, O, Ca, and P, consistent with published literature on recovered wear particles^8,20,22,42^. Sample B indicated disproportionately greater Co release into solution. XRD analysis of the surface suggested the alteration of crystallographic structure of the CoCrMo alloy.

Sample C, representing samples subjected to a 25 MHz, 200 mVpp square wave, displayed generalized surface deposition. The EDS analysis illustrated mixed deposition of Cr, C, O, Ca, P, N, Na, and Cl. Excitation with 25 MHz and 75 MHz harmonics within the square wave resulted in increased surface deposition and decreased metal ion release into solution.

Therefore, it is believed that the presence of oscillatory electric fields surrounding an implant, may manipulate and/or accelerate the production of chemical species formed at the surface of the metal alloy. These results indicate that the electromagnetic environment surrounding implanted metal alloys may affect their corrosion properties. Future study and classification is required to develop a fundamental understanding of this electrochemical phenomenon. Current literature does not adequately predict the effects of non-ionizing, oscillatory electric potentials on the surface/fluid chemical interaction present on the surface of the prosthesis.

## Data Availability

All relevant data files, figures, and codes are available upon reasonable request.
